# Vertebrates used for medicinal purposes by members of the Nyishi and Galo tribes in Arunachal Pradesh (North-East India)

**DOI:** 10.1186/1746-4269-7-13

**Published:** 2011-03-31

**Authors:** Jharna Chakravorty, V Benno Meyer-Rochow, Sampat Ghosh

**Affiliations:** 1 Biochemical Nutrition Laboratory, Dept. of Zoology, Rajiv Gandhi University, Arunachal Pradesh 791112, India; 2 School of Engineering and Science, Jacobs University, Research II (Rm. 37) D-28759 Bremen, Germany

## Abstract

Arunachal Pradesh, the easternmost part of India, is endowed with diverse natural resources and inhabited by a variety of ethnic groups that have developed skills to exploit the biotic resources of the region for food and medicines. Information on animals and animal parts as components of folk remedies used by local healers and village headmen of the Nyishi and Galo tribes in their respective West Siang and Subansiri districts were obtained through interviews and structured questionnaires. Of a total of 36 vertebrate species used in treatments of ailments and diseases, mammals comprised 50%; they were followed by birds (22%), fishes (17%), reptiles (8%) and amphibians (3%). Approximately 20 common complaints of humans as well as foot and mouth disease of cattle were targets of zootherapies. Most commonly treated were fevers, body aches and pains, tuberculosis, malaria, wounds and burns, typhoid, smallpox, dysentery and diarrhoea, jaundice, and early pregnancy pains. Very few domestic animal species (e.g., goat and cattle) were used zootherapeutically. More frequently it was wild animals, including endangered or protective species like hornbill, pangolin, clouded leopard, tiger, bear, and wolf, whose various parts were either used in folk remedies or as food. Some of the animal-based traditional medicines or animal parts were sold at local markets, where they had to compete with modern, western pharmaceuticals. To record, document, analyze and test the animal-derived local medicines before they become replaced by western products is one challenge; to protect the already dwindling populations of certain wild animal species used as a resource for the traditional animal-derived remedies, is another.

## Introduction

Scientific research is revealing an ever increasing number of links between biodiversity and human health, not only in terms of food resources or food security, but also with regard to materials to treat and cure diseases. Since ancient time plants and animals, or parts of them, have been used therapeutically and even today animal and plant-based medicines continue to play an essential role in world health care [1]. Although plant and plant-derived materials have received considerably more attention from scientists and are more commonly used in traditional medical systems than animal-derived products, the latter also constitute an important element in the *materialia medica*. In fact, the use of animals for medicinal purposes is part of a body of traditional knowledge, which is becoming more and more relevant to discussions on mammalian relationships and phylogeny [2], conservation biology, biological prospecting, and patenting [3-6]. It has been reported that more than half of the world's modern drugs are of biological sources [7,8] and that of the 252 chemicals that have been selected by the WHO as essential to human health, 8.7% come from animal sources [7].

It is fair to say that animals have been playing a significant role in healing processes, folk rituals, and religious practices of peoples from all five continents [6,9-12]. In traditional Chinese Medicine more than 1500 animal species have been recorded to be of some medicinal use [13,14]. A list of 60 different species of insects used to treat a wide range of disabilities and illnesses in Japan has been published [15] and 24 animal species were identified, whose by-products were used therapeutically by the Tamang people of Nepal [16]. In Pakistan, 31 animal-derived substances were said to constitute 9% of the total of the medicinal substances in the inventory of traditional healers [17]. Alves [18] conducted a study to review traditional treatments of a variety of ailments in North-East Brazil and recorded 250 animal species used in this context and Alves *et. al. *[19] reported that at least 165 reptile species were used in traditional folk medicines around the world.

In India, since times immemorial, investigations focused on various zootherapies and traditional medicines, documented in the ancient texts of the *Ayurveda *and *Charaka Samhita*. Because of its variety in geographic and climatic conditions, India is blessed with diverse flora and fauna, different tribal and ethnic communities, a multitude of cultural complexities. This rich diversity of traditional life styles and biological resources in the different states has permitted gathering together a wealth of ethnozoological knowledge. Yet, the documents containing these diverse pieces of ethnozoological information have been very fragmentary, so that Mahawar and Jaroli [20] conducted a review in which they documented approximately 109 animal species used in the treatment of different kinds of ailments in the whole of India. In another study, but restricted to the adjoining areas of the wild life sanctuary of Mount Abu, 24 animal species were reported to be of medicinal use [21]. Their investigation highlighted the variety of zootherapeutic uses among the tribes of India, especially those of Rajasthan, Maharashtra, Kerala, Andhra Pradesh, parts of Assam and Nagaland. Local uses of amphibians by inhabitants of the Arun Basin [22] and traditional zootherapeutic treatments among the tribal population of Tamil Nadu [23] were reported and ethnomedicinal uses of fish and other aquatic animals are known from Bangladesh [24]. Mishra et al [25] very recently described zoomedicinal uses from Orissa locals that involved animal parts of 7 species of vertebrates to treat 12 different illnesses. Work on the ethnic people of Arunachal Pradesh, however, has till now received only very scant attention (e.g., [26,27] and is in dire need of supplementary information.

Although traditional treatments, making use of animals or animal parts, have often been considered mere superstition, their persistence over hundreds or even thousands of years ought to be sufficient incentive to probe whether or not they are effective. And sure enough, the potency of at least some traditional medicines based on animals cannot be denied, since numerous such medicines have been methodically tested by pharmaceutical companies and turned into sources of drugs, which are now part of the armament of the modern healer [28]. For instance, peptides extracted from scraped secretions of *Phyllomedusa bicolor *(Amphibians), are used in the treatment of depression, stroke, seizures and cognitive loss in ailments such as Alzheimer's disease [29]. Early muscle relaxants were obtained from so-called poison arrow frogs, containing curare, a compound also used in psychiatric treatments [30]. The deer velvet extract pantocrin is nowadays marketed as a powerful antioxidant with anti-stress and immunomodulatory activity for use with humans [31] and animals, e.g. dogs [32].

Actually, while some uses of animals and their products as components of traditional medicines still remain unrecorded, the list of animals that can be used to obtain therapeutically important compounds from grows. Thus, we have a problem: the scholarly investigation of studies on the medicinal uses of animals and their products should not be neglected and ought to be considered a legitimate and important quest to complement the existing body of knowledge. On the other hand, species deemed most useful in this regard can easily be overexploited and become threatened by extinction [33].

The increasing relevance of ethnobiological knowledge across the globe and, on the other hand, the danger of losing this information before it can be properly documented, prompted us to embark on this study to record to what extent members of the Nyishi and Galo tribes of Arunachal Pradesh in the northeastern part of India make use of animals and their products in treatments of common ailments and diseases.

### Study Area and Methodology

**Figure 1:**
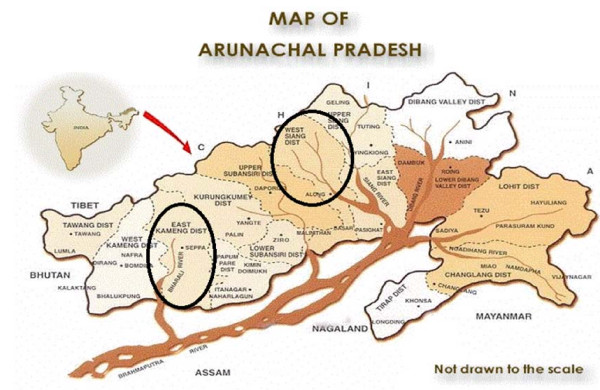
**Map of Arunachal Pradesh, showing study sites (for information on latitudes and longitudes, see Figs 1b,c)**.

**Figure 2:**
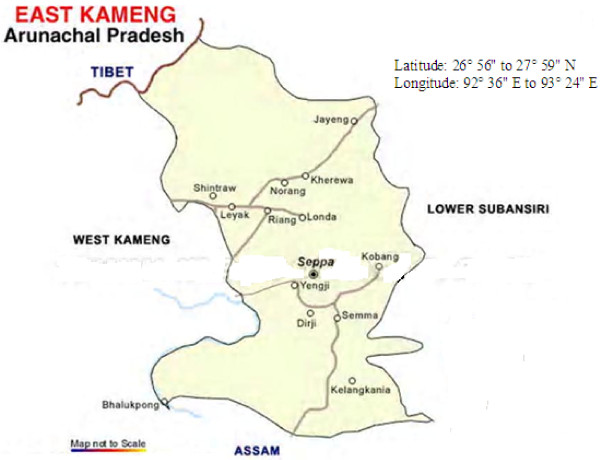
**Map showing East Kameng study site**. Adopted from: www.mapsofindia.com/maps/arunachalpradesh/districts/eastkameng.htm

**Figure 3:**
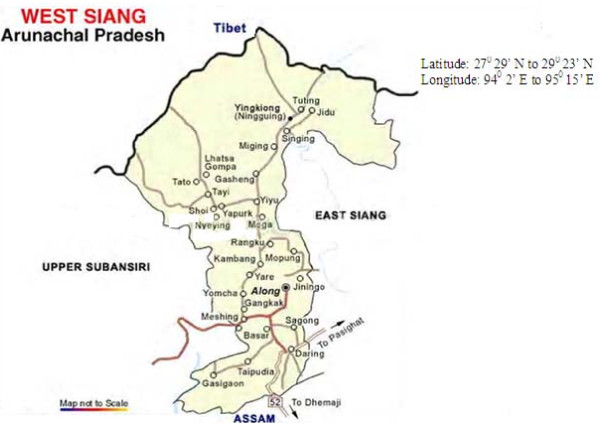
**Map showing West Siang study site**. Adopted from: www.mapsofindia.com/maps/arunachalpradesh/districts/westsiang.htm

Arunachal Pradesh (Figure 1) lies in the north-easternmost part of India and shares a major portion of the biological hot spot region of the Eastern Himalaya owing to its range of vegetation from tropical to alpine. The state is not only rich in floral and faunal diversity, but with 26 major tribes and 110 sub-tribes also in ethnic communities. Forests cover 60% of the total area of Arunachal Pradesh and the range of geographic, climatic, and cultural diversity has provided the backdrop for the wealth of traditional knowledge in this region of India. Traditional healing practices are one of the treasures of this resource-privileged region. The Nyishi and Galo tribes of Arunachal inhabit mainly the East Kameng (Nyishi) (Figure 2) and West Siang (Galo) (Figure 3) regions of Arunachal Pradesh, where literacy rate is relatively low, but life expectancy is high. Due to limited access to proper medical care and transportation, most of these people hold a traditional knowledge pertaining to the use of natural resources as medicines for community welfare. For their livelihood these tribal people are totally dependent on the forest and its resources.

As with our earlier study [34] data were obtained during visits to ten villages in each of the tribal areas, selected at random. The number of households per village was 12 - 20 (one village had 30). Frequently at least 2 houses were unoccupied, because the families had moved into the towns in search of work. At least two households per village, inhabited by village elders and their families, were visited. Recommendations by the headman or village elders to visit certain knowledgeable persons in another village were sometimes followed.

The interviewed people (20 persons aged between 45 and 70 years of age from each tribe) were asked simple questions like "how do you know when you are sick? What tells you that you are sick?". After having obtained a list of the major illnesses that the local people recognize and distinguish, we asked how they treated a person that suffered from such illnesses. When animals or parts of animals were involved, we requested that these animals be shown to us. In most cases the locals complied and with the help of illustrated identification guides [35-39] it was usually possible to identify the species in question. Where this was not possible, photographs of an animal in question were taken and later shown to an expert or compared with specimens in the university collection. To take voucher specimens back to the university was not possible for three reasons: firstly, many of the species involved are too large to be transported (e.g., goats, bulls, bears, porcupines etc); secondly, the locals would not let us take some of the specimens, fearing they could get into trouble; thirdly, many villages visited are so remote that no roads lead to them and one had to walk there, making transport of any material a very difficult undertaking. The vernacular names of the zootherapeutically (or otherwise important) vertebrates were written down phonetically, and notes were taken on the ailments treated with these animals, modes of preparation, assumed therapeutic value, related folklore and anything else considered worthwhile in connection with the species in question. As the knowledge of Hindi or English of the locals was often not great, our questions had to be simple and to the point. Further information was obtained from about 15 persons of Nyishi or Galo origin, regarded as knowledgeable, but living in the urban areas. Their selection as informants was based on the input provided by the villagers. According to the locals, their own knowledge of medicinal animals was acquired through parental heritage, or because they had experienced folk medicine healing their kin and/or themselves. Most people interviewed could come up with at least 8 different zootherapies. However, in order to obtain an idea on how widespread and common the particular zootherapeutic knowledge was, we decided, as with our earlier study [34] to only accept into our list animals and their products when at least 40% of the respondents answered in the same way.

## Results and Discussion

**Figure 4:**
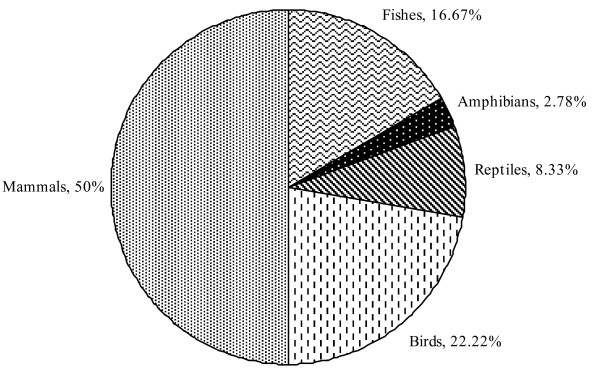
**Percentages of species in different vertebrate classes reported for medicinal use by Nyishi and Galo tribes of Arunachal Pradesh**.

We examined the traditional zootherapeutic uses that Nyishi and Galo people have for vertebrates and their parts in treating various kinds of diseases of humans and livestock. Table 1 summarizes the scientific names of the medicinally used vertebrates, their vernacular names, the part(s) of the animal used, the diseases or ailments the animal-derived medicines are thought to be effective for, and the ways the treatments are carried out. Table 2 summarizes the present conservation status of the vertebrates mentioned in Table 1 as zootherapeutically important. Altogether 36 species of vertebrates were identified to play a role in the treating humans and animals suffering from a variety of ailments and ills. Out of these 36 species, the use of mammals and their parts was highest, constituting about 50% (n = 18); next came birds (n = 8; 22.22%), fish (n = 6; 16.67%), and reptiles (n = 3; 8.33%). Amphibians were used least (n = 1, 2.78%) (Figure 4). In similar studies carried out around the world, mammals and birds also recorded the highest use as part of local folk medicines [19,20,40-47]. Surveys other than our own research from north-eastern India indicate the same [48,49]. However, for the tribal populations of the Garo hills in Meghalaya (NE India) Sharma and Khan [43] observed that drugs of insect origin were more common than those derived from vertebrates.

**Table 1:** Inventory of vertebrate species used for medicinal purposes by members of Nyishi (N) and Galo (G) tribes in Arunachal Pradesh (N E India)

	Common names	Vernacular name	Scientific name	Used by N or G	Parts used	Indication	Prescription	Uses elsewhere in India
**Fish**								

1	Eel	Ngub (G,N)	*Anguilla *sp.	N & G	Body mucus	Burns	Body mucus to be applied on burn areas of the body	Fresh blood is drunk to treat asthma and general weakness by Ao tribe of Nagaland [49].

2	Fresh water fishes	Ngui (N)	*Semiplotus *sp., *Labeo rohita*	N	Stomach & gut	Stomach ache & digestive problems	Intestines & stomach are smoked in fire, mixed with salt and taken with rice 2-3 times a day. Also taken as a preventive measure.	Cervical vertebra of *L. rohita *are used in urine blockage problem by the Saharia of Rajasthan [67].

3	Gangetic goonch	Nguri (G)	*Bagarius bagarius*	G	Fins, bones	Body burns, Stomach pain	Smoked dried bones/fins are burnt to ash and applied on burnt portion twice a day. A pinch of ash is taken along with water.	---

4	Catfish	Ngui (G)	*Amblyceps *sp.	G	Bones	Body burns.	The cooked fish bones are burnt to ash and applied to the burn or wound until healing is observed. The ashes can be preserved for further use.	---

5	Ballitora minnow	Ngoka ngui (N) Nyoka pagra (G)	*Psilorhynchus ballitora*	N & G	Whole body	Diarrhoea	Smoked, dried fish is eaten	---

6	Kingfish + earthworm	Ngui + tadar (N)	*Semiplotus *sp.+ *Pheretima *sp.	N	Whole body	Smallpox	Cooked with fish and fed to the children suffering from smallpox	---

**Amphibia**								

7	Frog	Taker (N)	*Rana *sp.	N	Whole body	Wound healing	Live crushed frog is applied to wounds from insect bites (must be carried out near fire place) twice a day.	Skin is used for wound healing by Ao tribe of Nagaland [49]. Flesh is used for wound healing by Irular, Mudugar, Kurumber of Western Ghat Kerala [60].

**Reptiles**								

8	Python	Burum (G,N)	*Python molurus*	N & G	Body fats	Massage for joint pain	Fats are stored in bamboo containers and used in body massage to cure joint pain.	Similar fat used for treatment of rheumatic pain, toothache by Irular, Mudugar, Kurumber tribes of Western Ghat Kerala [60]. But, fried meat is used to improve eyesight while snake's slough is used for cattle by Garasiya people of Rajasthan [21]. Fat is reported in treatments of leprosy by tribal populations of Tamil Nadu [61].

9	Cobra	Tabih (G)	*Naja *sp.	G	Flesh	1. Preventive 2. Foot and mouth disease of cattle 3. Magical	1. Cooked meat is taken as preventive measure for common diseases like colds, flues and epidemics. 2. Raw meat is crushed with little salt and fed to cattle suffering from foot and mouth disease. 3. Taking snake meet keeps away from evil spirits.	Meat is believed to improve eye sight & facilitates urination. Similar use in foot and mouth disease of cattle but tribes like Koya, Guthikoya, Lambada, Mala of Andhra Pradesh use skin unlike raw meat in [59]. Slough is used to decorate the home and as well in worship by Garasiya people of Rajasthan [21].

10	Monitor lizard	Horkek(G) Baminsopin (N)	*Varanus bengalensis*	N & G	Flesh	Cough, fever	Flesh boiled and taken whenever available as a preventive measure for coughs and fevers.	Meat promotes strength and vitality and fat used for joint pain by Koya, Guthikoya, Lambada, Mala tribes of Andhra Pradesh [59]. Skin and fat used for treating piles, rheumatism, body pain by Ao tribe of Nagaland [49]. Fat is used for massage to treat arthritis by Irular, Mudugar, Kurumber tribes of Western Ghat Kerala [60]. Cooked flesh is eaten by Garasiya people of Rajasthan to promote body stamina [21]. Oil is used for back pain [68]. Flesh is used to treat arthritis by tribals of Tamil Nadu [61].

**Birds**								

11	Hornbills: 1. Necked 2.Weathered 3. Great 4. Pied	Poe, Paga (N,G)	1. *Aceros nipalensis*, 2. *A. undulatus* 3. *Buceros bicornis*, 4.*Anthracoceros albirostris*	N & G	Fats, .	Body massage to ease body pains	Stored fats are commonly used for massaging aching body parts.	Cooked flesh is used for the treatment of rheumatic pain by Irular, Mudugar, Kurumber tribes of Western Ghat Kerala [60].

12	Crow	Pa (N) Pak (G)	*Corvus splendens*	N & G	Flesh	Stomach disorder	Dried meat is taken to minimize stomach upsets. Meat fed to children improves their intelligence.	Flesh is used for treatment of rheumatism, paralysis, earache by Ao tribe of Nagaland [49]. Fat is used to treat smallpox & malaria by Mompa tribe of Arunachal Pradesh [27]. Meat cooked in mustered oil is used for leucoderma by Irular, Mudugar, Kurumber tribes of Western Ghat Kerla [60]. Excreta are topically applied to cure blisters by Garasiya people of Rajasthan [21]. Flesh is traditional medicine for whooping cough by Kachch of Gujrat [69] and anaemia in tribals of Tamil Nadu [61].

13	Eagle	Kyokam (N)	*Spilornis cheela*	N	Fat and feathers	Burns, wounds body sprains	Oil applied locally and wounds covered by feathers.	Fat is used to treat sprains & burns by Ao Nagas [49], but malaria & typhoid by Arunachal Pradesh's Mompas [27].

14	Owl	Puptal (G)	*Bubo nipalensis B. bubo*	G	Flesh	Maleness (malevolency)	Smoked flesh is taken	Owls are of importance in the zootherapeutic treatments, but the species differ in different parts of the country. Similar use: Meat of *Strixaluca nivicola *(owl) promotes strength & vitality as used by Koya, Guthikoya, Lambada, Mala tribes of Andhra Pradesh [59] and Shoka people of Uttaranchal [70], but wings of *Otus bakkamoena *burnt and inhaled in order to reduce stomachache by Garasiya of Rajasthan [21].

**Mammals**								

15	Mithun	Sobo (G) Sebe (N)	*Bos frontalis*	N & G	Gall bladder, testicles.	1. Dysentery, Coughs & fever 2. Lactation of mother	1. Gall bladder is filled with rice powder and tied properly and smoked dry. A pinch of it is cooked with rice and taken until disease is cured. 2. A pinch of smoked, dry testes is cooked and fed twice a day to a mother who is secreting less milk than expected after delivery.	Penis is used to treat skin disease, breast pain of lactating mother by Ao tribe of Nagaland [49]; also reported from Arunachal Pradesh [26].

16	Goat	Sabing (N)	*Capra hircus*	N	Gall bladder & frontal bone.	Fever & early pregnancy pain, stomach ache	The frontal bone is burnt and taken in pinches mixed with boiled water 2-3 times a day to minimize fever and early pregnancy pain. Gall bladder is cooked with rice and taken for stomach ache.	Meat is reported to stimulate digestion among tribes like Koya, Guthikoya, Lambada, Mala of Andhra Pradesh [59]. Soup of leg bone is used to cure weakness; urine is used for tuberculosis by Saharia tribe of Rajasthan [67]. Urine of *Capra sibirica *is used to treat asthma by Ao tribe of Nagaland [49].

17	Rat	Kojak (N)	*Rattus *sp.	N	Whole body	To minimize pain after conception	Whole body is burnt and crushed or powdered, taken with rice as a painkiller after conception (early pregnancy).	---

18	Mole	Kor tab (N)	*Talpa *sp.	N	Flesh	Tuberculosis	Flesh/Meat is cooked and eaten in order to cure tuberculosis.	Flesh is used for asthma by Ao tribe of Nagaland [49].

19	Fox	Siyali (N)	*Vulpes bengalensis, Canis aureus*	N	Flesh	Tuberculosis	Meat is boiled or roasted and taken.	Fat is used for rheumatism, skin disease by Irular, Mudugar, Kurumber tribes of Western Ghat Kerala [60].

20	Wolf	Sarchi (N)	*Canis lupus*	N	Skin	Coughs & fevers, epidemics	Skin (whenever available) is burnt and taken in pinches as a preventive measure.	Meat is used to cure asthma, paralysis & arthritis by Koya, Guthikoya, Lambada, Mala of Andhra Pradesh [59].

21	Porcupine	Sihi (N) Hoi (G)	*Hystrix *sp.	N & G	Gall bladder, stomach & intestines, flesh	Diarrhoea, gastritis, tuberculosis	1. Gall bladder, stomach and intestines (whenever available) are boiled and taken with rice as a preventive measure for diarrhoea and gastritis. 2. The meat and stomach portions are cooked and fed to a person suffering from tuberculosis.	Similar use: Dried stomach & intestine used for digestive disorders by Koya, Guthikoya, Lambada, Mala of Andhra Pradesh [59]; bile for dysentery by Ao tribe of Nagaland [49]; boiled flesh for stomachache, piles, breathing trouble by Irular, Mudugar, Kurumber of Western Ghat Kerala [60]; boiled gut content is used to treat stomachache, indigestion and coughs and colds by tribals in Arunachal Pradesh [26].

22	Pangolin	Hosik(G)	*Manis pentadactyla*	G	Nails	Boils	For piercing the boils (assumed antiseptic property)	---

23	Mongoose	Sanf sakyo (N)	*Herpestes javanicus*	N	Whole body	Preventive measure for any disease	Roasted or boiled and taken as a preventive measure to avoid diseases.	Properly cooked penis is used to treat impotence by males of Ao tribe in Nagaland [49].

24	Deer	Hudum, hocher (G) Sudum (N)	*Moschus chrysogaster, M. moschiferus*	N & G	Gall bladder, fresh blood umbilicus	Malaria, diarrhoea, Fevers, stomach upset, body immunity, tuberculosis	Rice is cooked with fresh gall bladder and 100-200 g are taken once a day till some improvement is seen. A pinch of smoke dried umbilicus is mixed in 1/2 litre boiled water and fed to the patient till disease is cured (same prescription for all indications). Boiled blood is taken as food and considered to improve body immunity.	Similar: Musk is used to cure malaria, heart ailments and to promote immunity to lactating mothers by Koya, Guthikoya, Lambada, Mala of Andhra Pradesh [59]; malaria, diarrhoea by Mompa of Arunachal Pradesh [27].

25	Sambar deer	Hudum (G)	*Cervus unicolor*	G	Horn	Bursting open boils	Crushed horn particles added with very little salt and are used for bursting off boils.	Fat: massaged in cases of asthma & rheumatism by Irular, Mudugar, Kurumber tribes of Western Ghat Kerala [60]. Paste of antler to treat herpes by Saharia tribe of Rajasthan [67], the same prescription applied directly on the stomach by Garasiya people of Rajasthan for treatment of stomach ache [21]. Penis is used to treat hydroceles by tribal population of Tamil Nadu [61].

26	Moon bear Black bear	Hutum (G) Sutum (N)	*Ursus thibetanus, Selenarctos thibetanus*	N & G	Gall bladder	Malaria, diarrhoea, fever, stomach upsets, other common diseases, body immunity.	The bladder is filled with rice powder and smoke dried; a pinch is either mixed with rice or taken directly once a day, till the disease gets minimized. Dosage is same for all.	Similarly: Gall bladder of *Selenarctos thibetanus *is used for treatment of malaria, typhoid and other serious fevers by Mompa of Arunachal Pradesh [27]; same prescription to treat stomach ache and diarrhoea, in Arunachal Pradesh [26] and bile of *Selenarctos *is used to cure malaria by Ao Nagas [49].

27	Tiger	Pate (N)	*Panthera tigris*	N	Bone and marrow	Jaundice	Cooked into soup and fed to the patient.	Dried bones are used for treating rheumatic and other body pain by Mompa of Arunachal Pradesh [27]. Flesh and fat are used for treatment of leprosy by tribal population of Tamil Nadu [61].

28	Clouded leopard Common leopard	Hogya (N)	*Neofelis nebulosa, Panthera pardus*	N	Bone marrow	Body pains	Bone marrows are preserved in bamboo cups and used for body massaging	Fat is used as massage for body pain by Koya, Guthikoya, Lambada, Mala tribes of Andhra Pradesh [59] instead of bone marrow. Flesh is used for treating typhoid, malaria, rheumatic pain by Mompa of Arunachal Pradesh [27].

**Table 2:** Present conservation status of animals mentioned in Table 1 and the paper (according to IUCN 2010 Red list of Threatened Species Version 2010.4)

Species	Status	Remarks
**Pisces**:	Least Concern Ver 3.1	

*Anguilla bengalensis *(Gray, 1831)[Synonym: *Muraena bengalensis *Gray, 1831]		

*Semiplotus *sp.	Data Deficient Ver 3.1	The specimen was not identified up to species level. In the place four species has been recorded *Semiplotus cirrhosus, S. manipurensis, S. modestus (*Burmese Kingfish) and *Cyprinion semiplotum *(Assamese Kingfish) [Synonym: *Cyprinus semiplotus*]. The present status for all except *C. semiplotum *is data deficient, Ver 3.1; for *C. semiplotum *Vulnerable, Ver 3.1

*Labeo rohita *(Hamilton, 1822)	Least Concern Ver 3.1	

*Bagarius bagarius *(Hamilton. 1822)	Near Threatened Ver 3.1	

*Amblyceps *sp.	--	The specimen could not be identified to species level

*Psilorhynchus balitora *Hamilton 1822	--	

**Amphibia**: *Hoplobatrachus tigrinus *(Daudin, 1802) [Synonym: *Rana tigrina *Daudin, 1802]	Least Concern Ver 3.1	

**Reptilia**: *Python molurus *(Linnaeus, 1758)	Lower Risk/Near Threatened Ver 3.2	

*Naja *sp.	--	The specimen was not identified to species level. However two species of *Naja *have been reported, *Naja kaouthia *(Monocled cobra) and *Naja oxiana *(Central Asian Cobra). For *N. kaouthia *the present status is Least Concern Ver 3.1 and for *N. oxiana *it is Data Deficient Ver 3.1

*Varanus bengalensis *(Daudin, 1802)	Least Concern Ver 3.1	

**Aves**: *Aceros nipalensis *(Hodsgon, 1829)	Vulnerable A2cd+ 3cd+ 4cd Ver 3.1	

*Aceros undulatus *(Shaw, 1811)	Least Concern Ver 3.1	

*Buceros bicornis *(Linnaeus, 1758)	Near Threatened Ver 3.1	

*Anthracoceros albirostris *(Shaw & Nodder, 1807)	Least Concern Ver 3.1	

*Corvus splendens *(Vieillot, 1817)	Least Concern Ver 3.1	

*Spilornis cheela *(Latham, 1790)	Least Concern Ver 3.1	

*Bubo nipalensis *(Hodgson, 1836)	Least Concern Ver 3.1	

*Bubo bubo *(Linnaeus, 1758)	Least concern Ver 3.1	

**Mammalia**: *Bos frontalis*		

*Capra hircus *(Linnaeus, 1758)	--	

*Rattus rattus *(Linnaeus, 1758)	Least Concern Ver 3.1	

*Talpa sp*	Least Concern Ver 3.1	

*Vulpes bengalensis *(Shaw, 1800)	Least Concern Ver 3.1	

*Canis aureus *(Linnaeus, 1758)	Least Concern Ver 3.1	

*Canis lupus *(Linnaeus, 1758)	Least Concern Ver 3.1	

*Hystrix sp*	--	The specimen was not identified to species level.

*Manis pentadactyla *(Linnaeus, 1758)	Endangered A2d+ 3d+ 4d Ver 3.1	

*Herpestes javanicus *(E. Geoffroy Saint- Hilaire, 1818) [Synonym: *Herpestes palustris *Ghose, 1965]	Least Concern Ver 3.1	

*Moschus chrysogaster *(Hodgson, 1839) [Synonym: *Moschus sifanicus *Buchner, 1891]	Endangered A2cd Ver 3.1	

*Moschus moschiferus *(Linnaeus, 1758) [Synonym: *Moschus sibiricus *Pallas, 1779]	Vulnerable A2d + 3d+ 4d Ver 3.1	

*Rusa unicolor *(Kerr, 1792) [Synonym: *Cervus unicolor *Kerr, 1792]	Vulnerable A2cd+ 3cd+ 4cd Ver 3.1	

*Ursus thibetanus *(G. [Baron] Cuvier, 1823) [Synonym: *Selenarctos thibetanus*]	Vulnerable A2cd+ 3d+ 4d Ver 3.1	

*Selenarctos thibetanus*		

*Panthera tigris *(Linnaeus, 1758)	Endangered A2bcd+ 4bcd+ C1+ 2a(i)Ver 3.1	

*Neofelis nebulosa *(Griffith, 1821)	Vulnerable C1+ 2a(i) Ver 3.1	

*Panthera pardus *(Linnaeus, 1758)	Near Threatened Ver 3.1	

**Figure 5:**
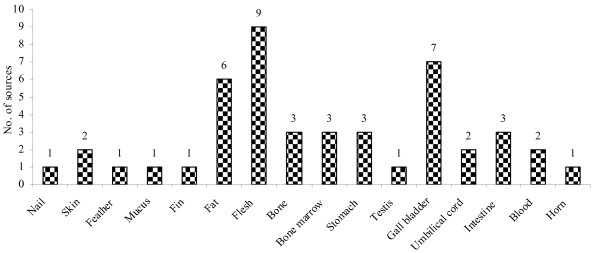
**Raw materials derived from animals used as therapeutic agents by members of the Nyishi and Galo tribes of Arunachal Pradesh**.

Of the zootherapeutic species recorded in the present study either whole animal bodies, body parts, or the animals' products were used. Zootherapeutic animal body parts or their products were assigned to one the following 16 categories of raw materials that formed the basis of medicines and were prescribed for treating locally diagnosed ailments. The 16 categories were: 1. claws and nails, 2. skin, 3. feathers, 4. mucus, fins, 6. fat, 7. flesh, 8. bone, 9. bone marrow, 10. stomach, 11. intestine, 12. testes, 13. gall bladder, 14. umbilical cord, 15. blood, 16. horns and antlers. Frequently the sought-after body parts did not always have to come from the same species. For example gall bladders from seven different species were assumed to be of therapeutic value (Figure 5). Some of the animal-derived medicines and preserved animal body parts are sold at the local traditional tribal markets. Collecting the raw materials involves manual gathering, slaughtering of livestock, or hunting and killing of wild species. Modes of preparation and administration of the animal-based medicines are presented in Table 1.

### Types of diseases

**Figure 6:**
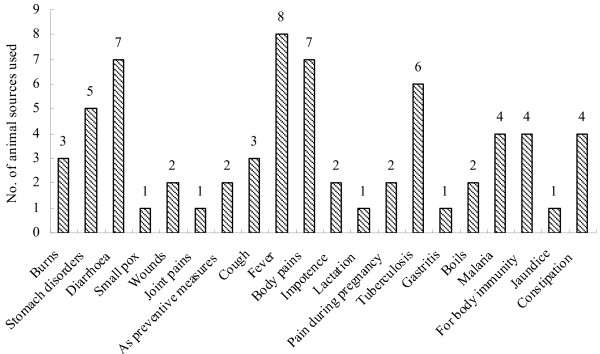
**Number of medicinal uses reported in connection with different indications amongst members of the Nyishi and Galo tribes of Arunachal Pradesh**.

About 20 types of common human ailments/diseases (and foot and mouth disease of cattle) were said to be curable by using some of the aforementioned animal materials (Figure 6). Conditions most widely subjected to animal-derived treatments were fevers, body pains and pains of the joints, diarrhoea, tuberculosis, stomach disorders, constipation, malaria, burns, coughs, wounds, typhoid, smallpox, dysentery, jaundice, stomach ache. The two ethnic groups under study seemed to know only the most common ailments encountered in day-to-day life. It was also observed by the locals that the treatment of one disease could have an effect, either positive or negative, on other diseases and that body parts of different animals could exert similar effects. To be specific, treatment of tuberculosis, for instance, could involve body parts of any one of these animals: porcupine, deer, fox, or mole. Using a variety of remedies for one and the same ailment and then finding that one of them turns out more potent than the others is a popular strategy [50]. It can lead to the adoption of a particular animal or animal part (depending on availability or accessibility) [51] in connection wit a specific condition. Given the fact that hundreds of plants assumed to possess medicinal effects have been identified from North-East India [52,53], one can assume that treatments solely based on animals or animal products are rare and that treatments involving animal material will frequently contain a plant component as well. The role of plants, however, has not been a topic of this study and therefore remains uninvestigated.

### Preparation and administration

Although distinct preparation and administration methods of the zootherapeutic resources existed (Table 1), some generalities were also noticed. For example, the fats of very different animal species like python, hornbill and eagle, to name but a few, is always heated up and then externally applied to relieve pain. Body parts of most species are either cooked, crushed into powder or boiled and then eaten. The use of flesh is common and usually taken in cooked or smoked form. Gall bladders and their contents seem another important raw material for members of both tribes. Beyond the uses for treating human diseases, zootherapeutic resources are also employed in ethno-veterinary medicines, e.g., for the treatment of foot and mouth disease in cattle. Barboza *et. al. *[54] even described zootherapeutic uses in connection with treatments of wild animals.

The relatively large number of medicinally important vertebrate species catalogued, demonstrates the importance of zootherapeutic practices as an alternative to newly introduced western medicines amongst the Nyishi and Galo tribes. Of the 36 identified medicinal animal species, many are also, at least occasionally, used as food. This high percentage of animal species with such twin function as food and medicine is not surprising, given the important role that wildlife as a source of protein plays for the local inhabitants. Similar cases, in which food animals were also used in remedies, were reported from other parts of the world [18]. Our knowledge of the criteria used by the tribals to decide whether a species is primarily to be used as food or as part of therapies, however, is limited as a variety of tribal dietary taboos can obscure the information volunteered by an informant [55-57].

The use of animals for therapeutic purposes not only in remote but certain urban areas as well (often those occupied by the economically disadvantaged), suggests that zootherapeutic practices may function as a social conduit, aiding ethnic identity and cohesion amongst members of the Nyishi and Galo tribes. However, as elsewhere observed with regard to indigenous peoples and their traditional food systems [58], we also noticed that younger members of both tribes were more and more inclined to accept modern over traditional medicines.

### Inter-tribal comparisons

**Figure 7:**
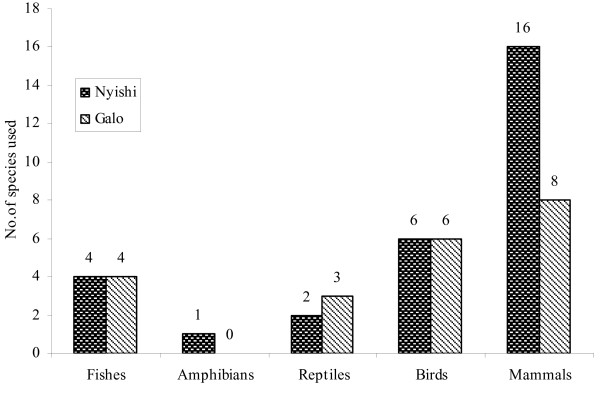
**Numbers of species in different vertebrate classes used for medicinal purposes by Nyishi and Galo tribes of Arunachal Pradesh**.

Our study revealed a difference between Nyishi and Galo people in the use of vertebrates for medicinal purposes (Figure 7). Nyishis use more often mammalian species than Galo do. Generally speaking, selectivity is a very complex issue, which brings several aspects into consideration when one compares the two tribes, e.g., differences in the availability of the animal-derived product(s), differences in motivation to go and obtain the product(s) in question, environmental factors like climatic and geographic differences, different agricultural practices and traditions, and differences in the prevalent disease spectrum. For the moment, therefore, we are unable to state anything more other than that differences between neighbouring tribes regarding species considered therapeutically valuable, would spread the pressure on the resource across several species, rather than focusing it on one alone. The same conclusion was reached by Meyer-Rochow [57] for situations, in which one species, but not another, was considered taboo by one tribe, but the same species, but not the other, was considered perfectly acceptable by a neighbouring tribe.

Some of the animal species used therapeutically by Nyishis and Galos are also used in very similar ways by a number of other ethnic groups in India. The porcupine, for example, supplies Nyishis and Galos with flesh that is used to treat individuals suffering from tuberculosis. The porcupine is also used by tribes of Andhra Pradesh, Kerala, and Nagaland to remedy upsets of the digestive system, but the specific raw materials from this very same animal differ: in Andhra Pradesh one uses the dried stomach [59], Kerala tribes boil the flesh and consume it [60] and Ao Nagas use the intestine, gallbladder and bile [49]. Members of the Ao Naga and the Mompa of Arunachal Pradesh also use the bile and gallbladder of a bear, but not just for digestive disorders, but also for fighting malaria attacks [27,49].

Musk deer flesh is used for enhancing body immunity and resistance to malaria by the Koya and Lambada tribes of Andhra Pradesh, the Ao Naga of Nagaland, and the Mompa of Arunachal Pradesh [27,59]. Amongst the tribes of Kerala hornbill fat is used to relieve body pain, but Nyishis and Galos use the bird's cooked flesh specifically to ease rheumatic pain [60]. Frogs (*Rana *spp.) are used by members of Nagaland and Kerala tribes to speed up wound healing, either through the consumption of whole frog bodies, the amphibian's flesh, or its skin alone [49,60]. The fat of the python also plays a role in the treatment of body pains or rheumatism amongst the tribes of Kerala [60], but peoples of Andhra Pradesh and members of the Nyishi and Galo use snakes in treating cattle that suffer from foot and mouth disease [59].

Depending on the region of India, some animals and their products can be put to very different uses. For example, the flesh of various species of monitor lizard is in use for treating humans suffering from coughs and fever by the Nyishi and Galo, but tribes of Andhra Pradesh, Kerala, and the Ao Naga of Nagaland use the same material to improve the overall vitality of a person and the fat of this reptile to treat rheumatism and pains of the joints [49,59,60]. The flesh of the crow is used in connection with stomach disorders by the Nyishi and Galo, but the same material is used in connection with rheumatism and paralysis by the Ao Naga [49] and with leucoderma by the tribes of Kerala [60].

The Mompa of Arunachal use the fat of the crow in cases of smallpox and malaria [27]. Members of the Nyishi and Galo tribes use the gall bladder of the mithun (*Bos frontalis*) in potions to stop dysentery, cough, and fever and prescribe the bull's testes to ease lactation problems of young mothers. Amongst the Ao Naga of Nagaland it is the bull's penis that is used for skin disorders and chest pain of lactating mothers [49]. The flesh of the fox, given to Nyishi and Galo children, is supposed to turn the children into cunning adults, but the flesh is also used as a tuberculosis remedy amongst the Nyishi. Tribes of Kerala employ the fox' fat in treatments of rheumatism and skin diseases [60]. Rather similarly, a wolf's burnt skin is taken to avoid attacks of cough or fever by the Nyishi, but the wolf's meat is used for alleviating asthma attacks, paralysis, and arthritis by the tribes of Andhra Pradesh [59]. An entire mongoose, eaten roasted or boiled, is said by Arunachal tribes to serve as a preventive measure of any disease, but its penis alone is used to treat impotence amongst the Ao Naga [49]. Crushed antlers of the sambar deer are used by the Galo for bursting open boils and the deer's fat is used as an asthma and rheumatism remedy by Kerala tribes [60]. Tiger bone is used for jaundice by the Nyishi, but according to members of the Mompa tribe dried tiger bone powder is said to ease rheumatic pains [27], while tiger flesh and fats are used for treatments of leprosy by tribal people of Tamil Nadu [61].

The use of hornbill species, thought by members of the Nyishi and Galo tribes to speed up healing processes, has not been reported earlier in India, although hornbills are regarded as medicinal by members of the Irular, Mudugar and Kurumbar tribes of the Western Ghats of Kerala [60]. Except for the Ao Naga of Nagaland [49] and the Nyishi and Galo of Arunachal Pradesh the use of the mithun (*Bos frontalis*) in traditional remedies has also not been reported from any other tribe in India as were the therapeutic uses of some freshwater fishes like, *Bagarius bagarius *and *Amblyceps *sp., the rodent *Rattus *sp., and the pangolin *Manis pentadactyla*. One reason could be the unavailability of certain species in a particular region; another would be lack of appropriate ethnobiological and ethnomedical field data.

### Zootherapies: impacts on society and environment

It is widely accepted that the use of certain plants and animals and their products in folk or traditional medicines indicates the presence of biologically active constituents in them. Although considerable information is available on phytochemistry and/or phytopharmacology of many traditional medicinal plants, bio-scientific evaluations of animal remedies are still quite rare [62]. Yet, some of the animals that are therapeutically used by traditional healers have been methodically tested by pharmaceutical companies and were, indeed, found to contain substances useful for the manufacture of drugs used in modern medicine [63].

The zoo-therapeutic knowledge, especially that of the Nyishi and Galo, was found to be based on few domestic and several wild animals, but some of the latter, serving as important folk medicinal resources like the hornbill, pangolin, clouded leopard, tiger, bear etc, are rare and protected species - a fact that is of considerable concern. Since Nyishis and Galos as well as other tribes have been using these animals for a long time, suppression to use them is not likely to save them from extinction. Rather it will be better to develop a conservation strategy, which is applicable to a particular locality giving the utmost respect to the ethnic sentiment and social structure of the locals. In accordance with Kunin and Lawton [63], those species directly involved in traditional medicines should be amongst those of the highest priority for conservation. According to Costa-Neto [33] research on zootherapies should be compatible with the welfare of the medicinal animal species, and the use of their by-products should be done in a sustainable manner. The species could be conserved through the integrated approach of in-situ and ex-situ conservation.

The establishment of a "Village Traditional Knowledge Bank" could be one of the significant approaches to not only conserve the diversity and related knowledge, but also to contribute in assuring quality of the livelihood of the ethnic people of Arunachal Pradesh in a broader sense and that of the Galo and Nyishi tribes in particular. The traditional medicines and the animal products used in the therapies should be tested for their effectiveness and chemical components; local folk should be made aware of the protected and endangered animal species and their importance as a resource for traditional medicines as well as for the region's tourist potential and biodiversity. Searching out or identifying substitutes for the medicinally used animal species should accompany the efforts of conservations. As has been suggested earlier [64,65] botanical alternatives to the use of the threatened animals should be considered. Moreover, it might be possible to replace the currently medicinally-used wild species with domesticated animals and their products, provided one can demonstrate similar therapeutic effects of the latter to the former. Thus, economic as well as ecological aspects need to be addressed [66].

## Conclusion

The main reasons for the popularity of zootherapies seem to be: a) economic and geographic accessibilities of animals assumed to possess therapeutic properties; b) the treatment's perceived efficacy; and c) socio-cultural factors like traditions, peer-pressure, cost of treatment, etc. Because Arunachal Pradesh is highly heterogeneous socially and profoundly unequal in the distribution of wealth and education, socioeconomic aspects clearly play a role in the persistence of zootherapeutic practices. Yet, the inclination of the younger generation towards welcoming and accepting modern medicine, while neglecting their own traditional body of knowledge with regard to the multitude of zootherapeutic uses, has cast considerable concerns on cleanliness, appropriateness, and effectiveness of the zootherapeutic products.

Hygienic conditions of zootherapeutically employed products are, indeed, generally poor and there is a need for sanitary testing and monitoring of animal-derived medicinal products. Additionally, chemical and pharmacological and perhaps epidemiological studies are necessary to clarify the eventual therapeutic usefulness of this class of biological remedies. Investigations of this kind would facilitate decisions on whether or not certain zootherapies could be accepted into public health programs. Finally, research into the abundance and availability of those particular animal species that are primarily used in the local therapies would be important in safeguarding them as a resource and, at the same time, in assuring their continued presence within the biodiversity of the region.

## Competing interests

The authors declare that they have no competing interests.

## Authors' contributions

JC carried out the field work and supervised SG's research. SG participated in the field work and identification of the insects. VBM-R began his ethnobiological studies in North-East India in 1991 and participated in the design, coordination, and draughting of the final manuscript. All authors read and approved the final manuscript.
